# Editorial: Vetinformatics: an insight for decoding livestock systems through *in silico* biology

**DOI:** 10.3389/fvets.2023.1292733

**Published:** 2023-10-30

**Authors:** Jun-Mo Kim, Rajesh Kumar Pathak

**Affiliations:** Department of Animal Science and Technology, Chung-Ang University, Anseong-si, Republic of Korea

**Keywords:** *in silico* analysis, molecular docking, molecular dynamics simulation, multiomics, network analysis, next-generation sequencing, vetinformatics

Computers have become an integral part of our daily lives, and we are dependent on them for many things. For instance, research is almost impossible without computers. In the early 1970s, Paulien Hogeweg and Ben Hesper coined the term “bioinformatics” (1), which became an independent discipline after its significant role in the Human Genome Project (HGP) (2). Decoding problems arising in the field of biological sciences via computation is known as bioinformatics (3).

With the world's population booming and natural resources dwindling due to human activity, veterinary science is becoming key in research and development to meet growing demands (4, 5). To meet the growing demand, veterinary science must integrate informatics to manage complex data and enhance research and development activities in various areas of veterinary sciences (4).

To enable novel discoveries, extensive use of *in silico* tools such as BLAST (6) and databases i.e. Bovine Genome Database, Porcine Translational Research Database, *etc* are required (6–8). Moreover, computers and information science have been integrated into all aspects of the veterinary profession, leading to the concept of vetinformatics. While bioinformatics is a broad field that focuses on several areas of biology, vetinformatics is specifically concerned with addressing problems in the field of veterinary science (4).

The establishment of the Association for Veterinary Informatics took place in 1981 (https://avinformatics.org/), where it was connected veterinary with informatics, but its focus is primarily on veterinary medicine. Some articles have also been published on “veterinary informatics”, where informatics has been used to advance the field of veterinary medicine (9, 10).

In 2016, Sujatha et al., published a brief review entitled “*Vetinformatics: A New Paradigm for Quality Veterinary Services*”. This review attempted to highlight applications of vetinformatics in veterinary science (11). Subsequently, in 2022 we authored a comprehensive review on vetinformatics, entitled “*Vetinformatics from functional genomics to drug discovery: Insights into decoding complex molecular mechanisms of livestock systems in veterinary science*” in which we covered many aspects (4). Accordingly, vetinformatics should also be considered as an important subject, similar to Pharmacoinformatics, Chemoinformatics, Genomeinformatics, Agriinformatics, Cropinformatics, Biomedical informatics and other informatics fields are considered. Therefore, vetinformatics is new and does not have an interesting history yet, but its approaches are important for problem solving not only in veterinary medicine but also in various areas of veterinary science (4).

In veterinary science, animal production is a highly intricate process with three basic interconnected components: animal biology, environment, and management techniques. Therefore, *in silico* approaches are required to bridge the gaps between genotype and phenotype to enhance efficiency in livestock productivity and sustainability. With the advent of several omics platforms and next-generation sequencing technologies, an enormous amount of animal data has been generated. While major bioinformatics databases and tools are available for the management and analysis of these data, veterinarians require animal and species-specific databases for effective management and future use. In addition, animal-specific tools for data analysis and integration, as well as computational and mathematical models for predicting the behavior of animal systems in different conditions, are necessary. Hence, vetinformatics has become an essential subject in the discipline of veterinary sciences, as it enables the handling and evaluation of large amounts of data and mining of important information that can aid researchers in decoding livestock systems to accelerate research and development.

Vetinformatics-related projects focus on the design and development of databases for the documentation of useful information about medicinal plants available in the literature and public domain for use in the discovery of herbal veterinary medicine. Animal genetic resource information is also being documented in the form of a database, and useful organism/species-specific databases are being created for the management of omics data sets. There is an effort to update the content available in veterinary databases for better functionality and create better and faster GUI-based data integration and analysis tools. Additionally, publicly available software is being improved for ease of use by veterinary biotechnologists and non-computer scientists and veterinarians. There is also a focus on the design and development of platform-independent software for vetinformatics research as well as vetinformatics training of undergraduate and graduate students and faculty in veterinary and animal science for analysis of multi-omics data.

The current Research Topic is “*Vetinformatics: An Insight for Decoding Livestock Systems Through In Silico Biology*”. Nine out of 17 articles were accepted for publication in this special Research Topic.

The first article in this Research Topic outlines the analysis of the structural and functional properties of *Mycoplasma gallisepticum* variable lipoprotein hemagglutin (vlhA) proteins, which are crucial for immune evasion. The results suggest diverse mechanisms for vlhA protein function in immune evasion, and the predicted 3D structure can aid in understanding its interaction with other molecules (Mugunthan and Harish). The second article reports the impact of preweaning vaccination on gene expression in calves. Results show that regardless of vaccination status, there was an increase in gene expression related to specialized proresolving mediator production, lipid metabolism, and stimulation of immunoregulatory T cells, while vaccination was associated with gene expression related to natural killer cell activity and helper T-cell differentiation (Scott et al.). The third article discusses the challenges facing the livestock industry due to climate change and increased demand for food and how new scientific and technological advancements can help. It highlights the importance of vetinformatics and its potential for improving veterinary research, breeding, disease prevention, management, and sustainability (Pathak and Kim). The fourth article outlines attempts to develop a multiepitope-based vaccine candidate using major and minor capsid proteins of infectious bursal disease virus. The proposed vaccine candidate has been evaluated as antigenic, immunogenic, and non-allergenic with potential to overcome the safety and protection issues of existing live-attenuated vaccines. Further experimental studies are required to assess the efficacy of the proposed vaccine candidate *in vivo* (Gul et al.). The fifth article suggests that the uncoupling proteins (UCPs) can be functional markers for identifying metabolic state, thermogenesis, and oxidative stress in birds, and their corresponding genes could be considered as candidates for use in breeding programs aimed at balancing energy expenditure and reactive oxygen species production (Davoodi et al.). The sixth article in this Research Topic aims to evaluate the quality of reference genomes and gene annotations in 114 species. The proposed next-generation sequencing (NGS) applicability index, which integrates 10 effective indicators, can help determine technological boundaries and examine the direction of future development in each species (Park et al.). The seventh article demonstrates the consistency and variability of data produced by reference-free *de novo* transcriptomes and reference-based datasets for identifying, annotating, and analyzing genes related to four major traits of water buffalo. The findings suggest that the characterized genes will enrich the knowledge of genetics for use in molecular breeding to improve the productivity of water buffalo (Mishra et al.). Articles eight and nine in this special Research Topic highlight the importance and application of machine-learning in veterinary science. They focus on detection of malignancies in canine subcutaneous and cutaneous masses (Dank et al.) and demonstrate the automated monitoring of diseased chickens (Bakar et al.).

Considering the current scenario and the increasing demand for *in silico* tools and databases for use in veterinary science, this series presents the achievements of vetinformatics and how it will be helpful in decoding livestock systems. This is a timely and exciting opportunity to harness the potential of vetinformatics for animal health and welfare.

## Author contributions

J-MK: Funding acquisition, Resources, Writing—original draft, Writing—review & editing. RKP: Writing—original draft, Writing—review & editing.
